# RATE OF CHANGE IN DEPRESSIVE SYMPTOMS OVER THE FINAL MENSTRUAL PERIOD

**DOI:** 10.1093/geroni/igad104.2030

**Published:** 2023-12-21

**Authors:** Nancy Avis, Alicia Colvin, Yuqing Chen, Hadine Joffe, Howard Kravitz

**Affiliations:** Wake Forest University School of Medicine, Winston-Salem, North Carolina, United States; University of Pittsburgh School of Public Health, Pittsburgh, Pennsylvania, United States; University of Pittsburgh School of Public Health, Pittsburgh, Pennsylvania, United States; Harvard Medical School at Brigham and Women’s Hospital, Boston, Massachusetts, United States; Rush University Medical Center, Chicago, Illinois, United States

## Abstract

Studies have shown that women are more vulnerable to elevated depressive symptoms during the menopause transition (MT) then before. This study evaluates change in depressive symptoms, as measured by the Center for Epidemiological Studies-Depression (CES-D) score, over the final menstrual period (FMP), a more precise measure of menopause timing compared to MT stages. The sample consisted of 1,783 women in the SWAN cohort with an observed FMP and at least one CES-D assessment from baseline through follow-up (1996 – 2017). Piecewise linear growth curves were fit to repeated measures of CES-D scores as a function of time from FMP (+/- 10 years). Results showed two places with a change in slope. CES-D scores decreased by 0.22 annually (95% CI: -0.41, -0.04) until 36 months prior to FMP (Period 1). From -36 months until the FMP (Period 2), there was a non-statistically significant increase in CES-D, followed by a decline (-0.19 annually, 95% CI: -0.34, -0.03) after FMP (Period 3). We examined a range of predictors (sociodemographic, health-related, psychosocial, hormonal) of change in CES-D score during each period. Upsetting life events were related to CES-D change at periods 1 and 2, social support at periods 1 and 3, and smoking at periods 2 and 3. Baseline CES-D was related at period 1, and anxiety was related to CES-D change at period 2. These results provide a more nuanced picture of change in depressive symptoms over the FMP and the variables related to depressive symptoms at different time points over the FMP.

